# Afatinib radiosensitizes head and neck squamous cell carcinoma cells by targeting cancer stem cells

**DOI:** 10.18632/oncotarget.15468

**Published:** 2017-02-18

**Authors:** Muzafar A Macha, Satyanarayana Rachagani, Asif Khurshid Qazi, Rahat Jahan, Suprit Gupta, Anery Patel, Parthasarathy Seshacharyulu, Chi Lin, Sicong Li, Shuo Wang, Vivek Verma, Shosei Kishida, Michiko Kishida, Norifumi Nakamura, Toshiro Kibe, William M Lydiatt, Russell B Smith, Apar K Ganti, Dwight T Jones, Surinder K Batra, Maneesh Jain

**Affiliations:** ^1^ Department of Otolaryngology/Head and Neck Surgery, University of Nebraska Medical Center, Omaha, NE 68198, USA; ^2^ Department of Biochemistry and Molecular Biology, University of Nebraska Medical Center, Omaha, NE 68198, USA; ^3^ Department of Internal Medicine, University of Nebraska Medical Center, Omaha, NE 68198, USA; ^4^ Department of Radiation Oncology, University of Nebraska Medical Center, Omaha, NE 68198, USA; ^5^ Department of Biochemistry and Genetics, Kagoshima University Graduate School of Medical and Dental Sciences, Kagoshima 890-8544, Japan; ^6^ Department of Oral and Maxillofacial Surgery, Kagoshima University Graduate School of Medical and Dental Sciences, Kagoshima 890-8544, Japan; ^7^ VA Nebraska Western Iowa Health Care System and University of Nebraska Medical Center, Omaha, NE 68198, USA; ^8^ Buffett Cancer Center, Omaha, NE 68198, USA; ^9^ Eppley Institute for Research in Cancer and Allied Diseases, University of Nebraska Medical Center, Omaha, NE 68198, USA

**Keywords:** head and neck squamous cell carcinoma (HNSCC), afatinib, radiosensitization

## Abstract

The dismal prognosis of locally advanced and metastatic squamous cell carcinoma of the head and neck (HNSCC) is primarily due to the development of resistance to chemoradiation therapy (CRT). Deregulation of Epidermal Growth Factor Receptor (EGFR) signaling is involved in HNSCC pathogenesis by regulating cell survival, cancer stem cells (CSCs), and resistance to CRT. Here we investigated the radiosensitizing activity of the pan-EGFR inhibitor afatinib in HNSCC *in vitro* and *in vivo*. Our results showed strong antiproliferative effects of afatinib in HNSCC SCC1 and SCC10B cells, compared to immortalized normal oral epithelial cells MOE1a and MOE1b. Comparative analysis revealed stronger antitumor effects with afatinib than observed with erlotinib. Furthermore, afatinib enhanced *in vitro* radiosensitivity of SCC1 and SCC10B cells by inducing mesenchymal to epithelial transition, G1 cell cycle arrest, and the attenuating ionizing radiation (IR)-induced activation of DNA double strand break repair (DSB) ATM/ATR/CHK2/BRCA1 pathway. Our studies also revealed the effect of afatinib on tumor sphere- and colony-forming capabilities of cancer stem cells (CSCs), and decreased IR-induced CSC population in SCC1 and SCC10B cells. Furthermore, we observed that a combination of afatinib with IR significantly reduced SCC1 xenograft tumors (median weight of 168.25 ± 20.85 mg; *p* = 0.05) compared to afatinib (280.07 ± 20.54 mg) or IR alone (324.91 ± 28.08 mg). Immunohistochemical analysis of SCC1 tumor xenografts demonstrated downregulation of the expression of IR-induced pEGFR1, ALDH1 and upregulation of phosphorylated γH2AX by afatinib. Overall, afatinib reduces tumorigenicity and radiosensitizes HNSCC cells. It holds promise for future clinical development as a novel radiosensitizer by improving CSC eradication.

## INTRODUCTION

Head and neck squamous cell carcinoma (HNSCC) is the sixth-most common cancer, accounting for over 600,000 new cases and 350,000 deaths worldwide per year [1]. Although primary HNSCC tumors are treatable, more than 50% of patients with locally advanced (LA) disease relapse, with either local recurrence (LR) or distant metastases associated with poor patient prognosis [2, 3]. Chemoradiation therapy (CRT) is the treatment of choice for LA HNSCC, but due to intrinsic tumor radioresistance [4], for decades patient prognosis has not improved [5–7]. While platinum-based CRT regimens have shown improved survival rates and locoregional control, these intensive regimens are associated with severe toxicities resulting in significant co-morbidities. Thus, for effective patient management there is an urgent need for the identification and development of novel agents to radiosensitize HNSCC tumors.

Epidermal growth factor receptor 1 (EGFR1), a member of the HER (ErbB) family of receptor tyrosine kinases that includes HER1/ErbB-1/EGFR, HER2/Neu/ErbB-2/EGFR2, HER3/ErbB-3/EGFR3 and HER4/ErbB-4/EGFR4, is over-expressed in a wide spectrum of tumors including ∼90% of HNSCCs [8, 9]. Over-expression of EGFR1 results in aggressive tumor behavior [10], radiation resistance [11], and poor prognosis [2]. EGFR family members by homo- or hetero-dimerization activate several downstream pathways, including Ras/Raf/MAPK/ERK, PI3K/Akt, STAT and the PLC-γ signaling pathways [12, 13] that potentiate the growth and survival of tumor cells and cancer stem cells (CSCs) [14]. The CRT regimen currently employed has been shown to activate EGFR signaling and to enrich and induce CSCs [15] leading to tumor recurrence [16].

Several preclinical and clinical studies have indicated the potential benefit of EGFR inhibition for radiosensitization of tumors and enhanced antitumor effects of CRT. However, recent studies have shown that the use of cetuximab or tyrosine kinase inhibitors (TKIs) like gefitinib, erlotinib and lapatinib result in development of therapeutic resistance and refractory disease by further upregulation/activation of HER2 and HER3 tyrosine kinases [17]. It is interesting to note that the use of dual anti-EGFR and HER3 antibody MEHD7945A has been shown to be more effective in inhibiting the proliferation of HNSCC cells *in vitro* and inhibiting the growth of xenografts tumors *in vivo* than cetuximab alone [18], suggesting that pan-EGFR inhibition could effectively inhibit or radiosensitize tumors and prevent recurrent tumors. Recently, the pan-EGFR inhibitor, afatinib, was also shown to reduce the CSC population in patient-derived leukemia cells, both *in vitro* and *in vivo*, by reducing their self-renewal [19].

Afatinib is a second generation FDA approved pan-EGFR-TKI that irreversibly binds to EGFR1, HER2 and HER4 [20, 21], and results in sustained inhibition compared to first-generation TKI inhibitors like gefitinib and erlotinib (reviewed in [22, 23]). Many preclinical and clinical studies have shown that afatinib significantly inhibits the growth of cancers that over-express either wild-type EGFR1 and/or HER2, or EGFR1 with L858R/T790M double mutations [22, 23]. Interim results of the phase III trial (LUX-Head & Neck1; NCT01345682) of refractory HNSCC patients have shown significantly improved, progression-free survival (PFS) with afatinib compared to methotrexate treated patients [24, 25]. In addition, *in vitro* studies using a single hypopharyngeal cell line FaDu have also shown that afatinib inhibits proliferation and enhances radiosensitivity [20, 21, 26].

In the current study, we examined the radiosensitizing effects of afatinib using *in vitro* and *in vivo* models of HNSCCs, and explored the underlying molecular mechanisms by which afatinib enhances radiosensitivity.

## RESULTS

### Afatinib and erlotinib inhibit the growth of HNSCC cells

**Figure 1:**
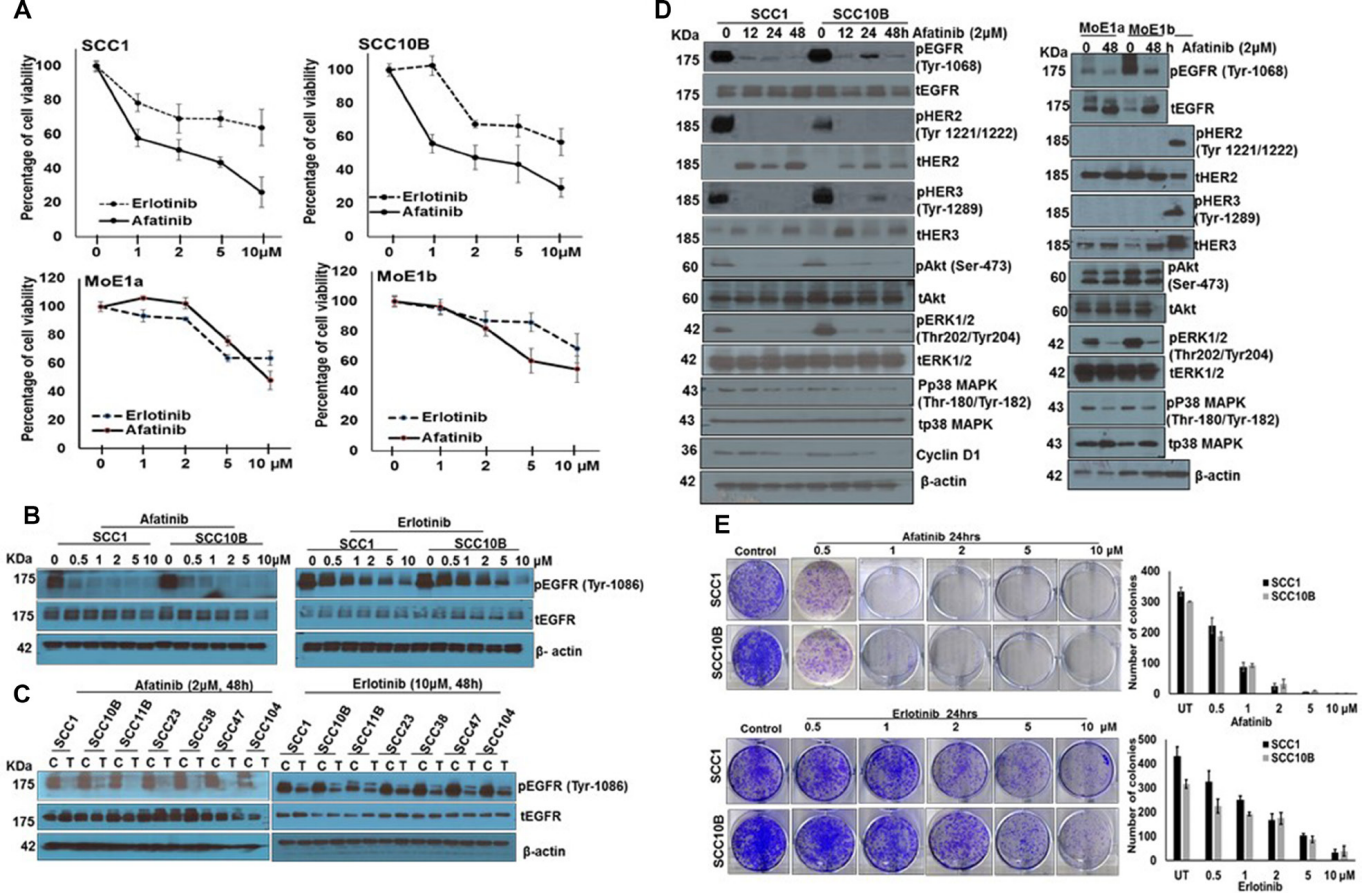
Afatinib and erlotinib differentially decreases the proliferation of HNSCC and normal cells (**A**) HNSCC cells SCC1 and SCC10B and immortalized normal oral epithelial cells MOE1a and MOE1b cells in 96 well plates were treated with different concentrations of afatinib and erlotinib for 48 h and viable cell number was analyzed by MTT assay. (**B**) Afatinib and erlotinib inhibits EGFR activation. SCC1 and SCC10B cells were treated with afatinib or erlotinib for 48 h and cell lysates were analyzed for pEGFR. (**C**) HNSCC SCC1, SCC10B, SCC11B, SCC23, SCC38, SCC47 and SCC104 cells were treated with either afatinib (2 μM) or erlotinib (10 μM) for 48 h and analyzed for pEGFR expression by Western blot analysis. β-actin was used as a loading control. (**D**) SCC1, SCC10B, MOE1a and MOE1b cells were treated with 2 μM of afatinib for 12–48 h and analyzed for phosphorylated and total forms of EGFR, HER2, HER3, AKT, ERK1/2, p38MAPK. Radiation (8 Gy)-treated SCC10B cells were used as positive controls for pHER2 and pHER3 expression in MOE1a and MOE1b cells. (**E**) Afatinib and erlotinib reduces colony formation of HNSCC cells. SCC1 and SCC10B cells were incubated with different doses of afatinib and erlotinib for 24 h and cells (1 × 10^3^) were seeded in triplicate in 10% DMEM in a 6-well plate. After 2 weeks, formed colonies were counted using the automatic colony counting tool by Quantity One Imaging software. The graphs represent the mean (± SE) number of colonies. The experiment was repeated twice (*p < 0.05).

To determine the cytotoxic effect of afatinib and to compare it with erlotinib, human HNSCC cells lines SCC1, SCC10B and normal oral epithelial cell lines MOE1a and MOE1b were treated with varying concentrations (1–10 μM) for 24–48 h (data shown for 48 h). MTT assay revealed dose- and time-dependent increase in cytotoxicity by both afatinib and erlotinib in SCC1 and SCC10B cells. Treatment with afatinib produced a cytotoxic effect with an inhibitory concentration at 50% (IC_50_) around 2 μM, whereas IC_50_ for erlotinib was around 10 μM. Both afatinib and erlotinib were less cytotoxic to MOE1a and MOE1b cells at their respective IC_50_ concentrations than SCC1 and SCC10B cells (Figure 1A). Moreover, Western blot analysis using anti-phosphorylated EGFR1 (pEGFR1 tyrosine-1068) antibody in afatinib- and erlotinib-treated SCC1 and SCC10B cells showed a dose-dependent decrease in pEGFR1, with more inhibition by afatinib compared to erlotinib (Figure 1B). However, no change in the total EGFR1 levels was observed in either afatinib- or erlotinib-treated cells, suggesting that afatinib is more effective in inhibiting EGFR signaling than erlotinib. In a panel of the HNSCC cell lines SCC11B, SCC23, SCC38, SCC47 and SCC104, we further observed that afatinib was more efficacious than erlotinib in inhibiting EGFR1 phosphorylation across all cells lines tested (Figure 1C).

### Afatinib differentially inhibits EGFR signaling in HNSCC and normal oral epithelial cells

We investigated the effect of afatinib on EGFR downstream signaling by treating HNSCC SCC1 and SCC10B cells with 2 μM for various time points. Western blot analysis showed significant inhibition of phosphorylation of EGFR1, HER2, and HER3 as early as 12 h after afatinib treatment in both cell lines (Figure 1D). Although there was no change in total EGFR1 levels, we observed decreased levels of total HER2 and increased levels of HER3 at 24 and 48 h of afatinib treatment, respectively. Consistent with the decreased levels of phosphorylated EGFR1, HER2, and HER3, we also observed significant deactivation of downstream signaling indicated by decreased levels of pAkt (Ser-473) and pERK1/2 (Thr202/Tyr204) (Figure 1D), but no significant change in levels of pP38 MAPK (Thr-180/Tyr-182). In contrast to the SCC1 and SCC10B cells, we observed low levels of pEGFR1 in immortalized oral MOE1b, but not in MOE1a cells, whereas pHER2 and pHER3 were undetectable in these cell lines. Although afatinib inhibited EGFR1 phosphorylation in MOE1b, we observed up-regulation of total EGFR1 and HER3 expression in both cell lines after afatinib treatment. The levels of total HER2 remained unchained (Figure 1D). Further, afatinib treatment decreased pERK1/2 levels in both MOE1b and MOE1a cells; however, in contrast to SCC1 and SCC10B cells, no change was observed in phosphorylation levels of Akt and P38 MAPK (Figure 1D). This differential phosphorylation of EGFR1, HER2, and HER3 in HNSCC and MOE1b and MOE1a cells, and the distinct effects of afatinib on downstream signaling pathways might explain its differential efficacy and toxicity on HNSCC and normal cells.

We compared the effects of afatinib and erlotinib on colony formation. Afatinib and erlotinib treatment resulted in significant inhibition of the colony-forming ability of SCC1 and SCC10B cells (Figure 1E), with afatinib more effective compared to erlotinib at each respective concentration (Figure 1E). At 2 μM (IC_50_), afatinib treatment resulted in a significant reduction of the number of colonies (*p* < 0.01), from 334 to 25 in SCC1 cells, from 300 to 33 in SCC10B cells (Figure 1E), while 10 μM concentration (IC_50_) of erlotinib reduced the number of colonies from 432 to 34 in SCC1 and 316 to 39 in SCC10B. Based on our MTT results, afatinib at 1–2 μM concentration was used for all subsequent experiments.

### Afatinib radio-sensitizes HNSCC cells

**Figure 2:**
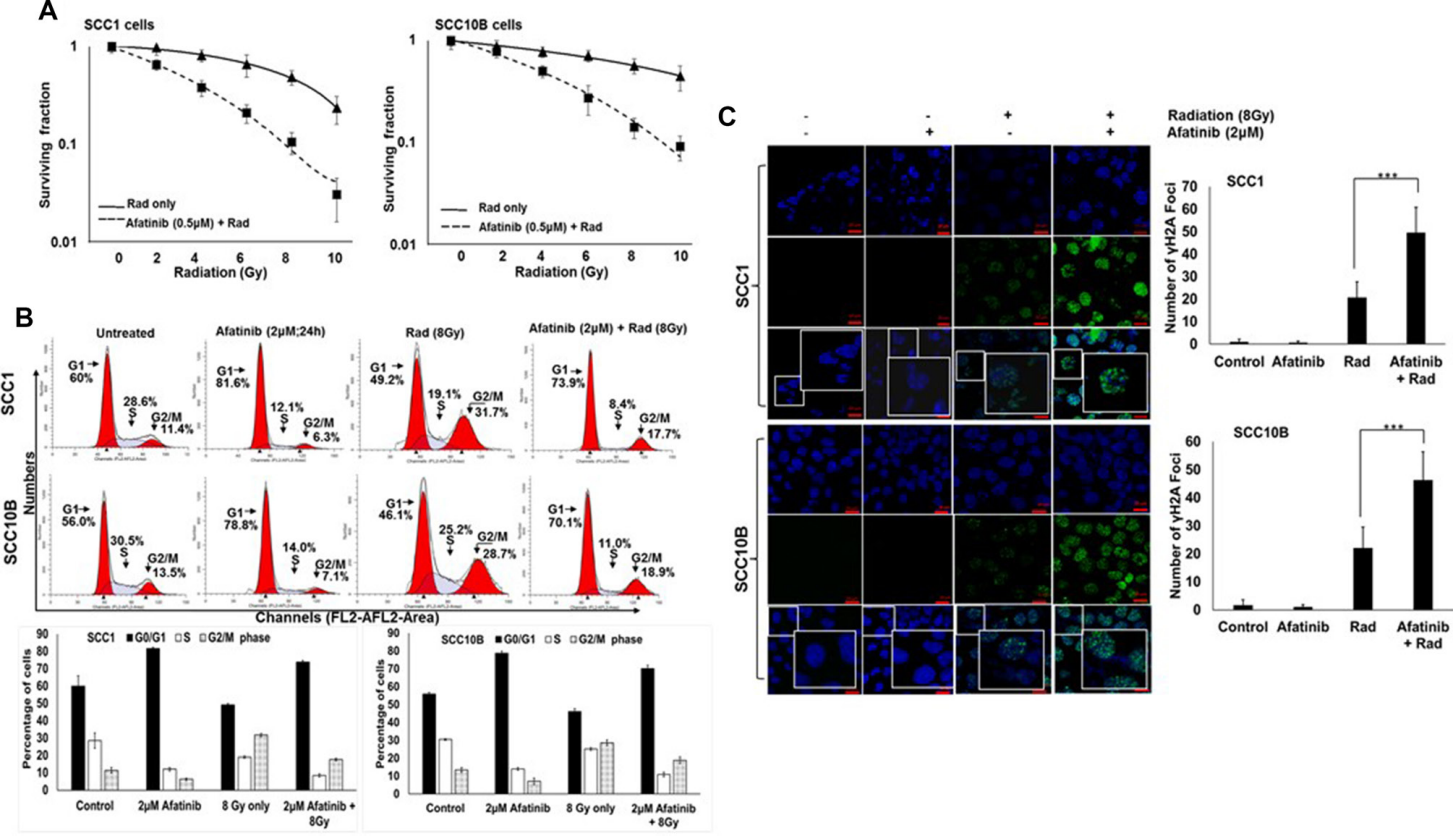
Afatinib radio-sensitizes HNSCC cells (**A**) SCC1 and SCC10B HNSCC were pretreated with afatinib (0.5 μM) for 24 h and then radiated with 2–8 Gy IR. After 24 h wells were washed and cells were allowed to grow for 2 weeks. Colonies were stained with 1% crystal violet, counted, and survival curves were plotted. (**B**) SCC1 and SCC10B cells were synchronized overnight in 1% serum containing medium and treated with afatinib alone for 24 h, or combined with 8 Gy IR. After 24 h, cells were fixed and stained with propidium iodide and analyzed by flow cytometry. (**C**) SCC1 and SCC10B HNSCC were plated on glass cover slips and pre-treated with afatinib (0.5 μM) for 24 h and then irradiated with 8 Gy IR. After 24 h wells were washed and analyzed by confocal microscopy for pγH2AX foci. DNA damage foci were counted and plotted as bar graphs.

As indicated in Figure 1D–1E, we observed that afatinib inhibited EGFR-mediated activation of Akt and ERK1/2, both of which are involved in radio resistance. To examine whether afatinib radio-sensitizes HNSCC cells, we performed a colony formation assay by pretreating SCC1 and SCC10B cells with 0.5 μM/L of afatinib 24 h prior to irradiation with 2, 4, 6, 8, or 10 Gy of IR. As shown in Figure 2A, while IR exposure alone induced a small decrease in clonogenic survival in both SCC1 and SCC10B cells, pretreatment with afatinib significantly decreased clonogenic survival of both SCC1 and SCC10B cells (Figure 2A). The sensitization enhancement ratio (SER), at a survival fraction level of 0.10, were 1.6 and 1.2 for SCC1 and SCC10B cells, respectively, after values were normalized to account for the effect of afatinib alone.

To understand the molecular mechanism of afatinib-induced radio-sensitization, SCC1- and SCC10B-treated cells were evaluated *via* flow cytometry following afatinib and radiation treatment; findings are summarized in Figure 2B. We observed that afatinib and ionizing radiation (IR) induced G_1_ and G_2_ cell cycle arrest respectively, with concomitant decrease of S phase in both SCC1 and SCC10B cells (Figure 2B). The mean percentage of G_1_ phase cells in untreated control cells and afatinib- and IR-treated cells were 60.0% ± 2.55%, 81.6% ± 2.32%, and 49.2% ± 3.19% for SCC1; and 56.0% ± 3.15%, 78.8% ± 1.45%, and 46.1% ± 2.21% for SCC10B cells. The mean percentages of S phase cells were changed in untreated control cells and afatinib- and IR-treated cells from 28.6% ± 1.35%, 12.1% ± 3.12%, and 19.1% ± 2.32%, and 30.5% ± 2.35%, 14.0% ± 2.35%, and 25.2% ± 3.11% in SCC1 and SCC10B cells, respectively. We then explored the cell cycle regulatory effects of afatinib when combined with IR in SCC1 and SCC10B cells. As shown in Figure 2B, pre-treatment with afatinib resulted in a further reduction in the S-phase and G_2_-M fraction compared to IR alone. The S phase decreased from 19.1% ± 2.32% to 8.4% ± 3.22% and 25.2% ± 3.11% to 11.0% ± 2.33% in SCC1 and SCC10B cells respectively. Similarly, G_2_-M fraction decreased from 31.7% ± 3.12% to 17.7% ± 2.32% and 28.7% ± 2.31% to 18.9% ± 2.14% in SCC1 and SCC10B cells, respectively.

No increase in the sub-G_1_ (apoptotic) fraction was observed in either of the cell lines investigated (Figure 2B), suggesting that neither afatinib alone, IR alone, nor the two combined induce apoptosis. Consistent with G_1_ cell cycle arrest by afatinib, we observed a time-dependent decrease in the expression of Cyclin D1 in both SCC1 and SCC10B cells (Figure 1D), which is involved in G_1_ - S phase transition. To further confirm the effect of afatinib pre-treatment on IR-induced DNA damage, we performed confocal microscopy for phosphorylated γH2AX foci; a marker for DNA double-strand breaks [27]. In both SCC1 and SCC10B cells, we observed increased pγH2AX foci per cell when treated with 8Gy IR, compared to almost none in the untreated cells (Figure 2C). The number of pγH2AX foci increased from 0.83 ± 0.51 and 1.66 ± 0.75 in untreated cells to 20.66 ± 2.86 (*p* = 0.0005) and 22.0 ± 3.09 (*p* = 0.0007) in IR treated SCC1 and SC10B cells, respectively. Afatinib pretreatment resulted in a further increase of the IR-induced pγH2AX foci to 49.5 ± 4.59 (*p* = 0.0004) and 46.16 ± 4.14 (*p* = 0.001) in IR treated SCC1 and SCC10B cells, respectively, suggesting that afatinib in combination with IR induces a defect in DNA repair machinery (Figure 2C). These observations suggest that afatinib pre-treatment attenuates IR-induced S and G_2_-M arrest, and that this can occur by affecting both the cell cycle regulatory proteins and DNA repair signaling pathways.

### Afatinib inhibits epithelial to mesenchymal transition in HNSCC cells

**Figure 3:**
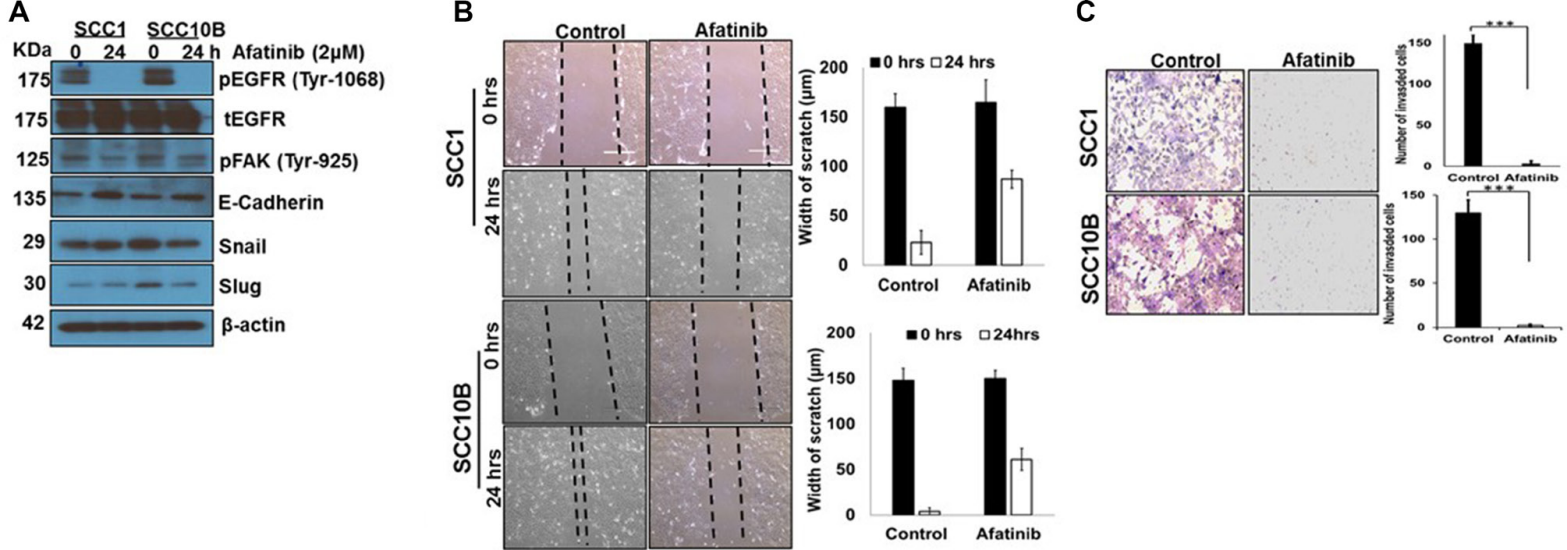
Afatinib inhibits Epithelial to Mesenchymal transition in HNSCC cells (**A**) SCC1 and SCC10B cells were treated with afatinib for 24 h and protein lysates were checked for pEGFR, pFAK, E-Cadherin, Snail, and Slug expression. β-actin was used as an internal loading control (**B**) SCC1 and SCC10B cells were allowed to form confluent layer in a 6 well plate and a scratch were made using a 200 μl sterile pipette tip. Unattached cells were washed with PBS and images were taken (t = 0 h). Cells were then treated with afatinib (2 μM) for 24 h and again photographed. The width of the wound was calculated with or without afatinib and bar graph plotted. (**C**) Afatinib treated and untreated SCC1 and SCC10B HNSCC cells (250 × 10^3^ cells) were seeded into the upper chamber of Matrigel gel coated Boyden chamber in serum-free αMEM media. Invading cells after 24h were stained using Diff Kit and quantified in 10 random fields under a light microscope (magnification, 100). Histograms represent mean of invasive cell number from three independent experiments; bars, SD.

Epithelial-mesenchymal transition (EMT) not only promotes invasion and metastasis, but also induces CRT resistance [28]. EGFR activation induces EMT, resulting in enhanced metastasis and resistance to cetuximab or IR in HNSCCs [29]. To understand whether afatinib radio-sensitizes HNSCC cells by modulating EMT, we analyzed the expression of EMT markers, cell migration (wound healing), and cell invasion (Matrigel based Transwell migration). Afatinib treatment resulted in increased expression of E-Cadherin in SCC1 and SCC10B cells, compared to control cells (Figure 3A), and, decreased the expression of Snail and Slug in SCC10B cells (Figure 3A). Treatment of cells with afatinib (2 μM) for 24 h significantly inhibited cell migration in SCC1 (*p* = 0.002) and SCC10B (*p* = 0.001) cells compared to controls (Figure 3B). In addition, afatinib also significantly inhibited the invasive potential of SCC1 (*p* = 0.004) and SCC10B (*p* = 0.003) (Figure 3C) compared to control cells. We also observed that decreased motility and invasion following afatinib treatment was associated with decreased FAK phosphorylation (Figure 3A) in both cell lines.

### Afatinib inhibits IR-induced DNA repair machinery and induces mitotic catastrophe

**Figure 4:**
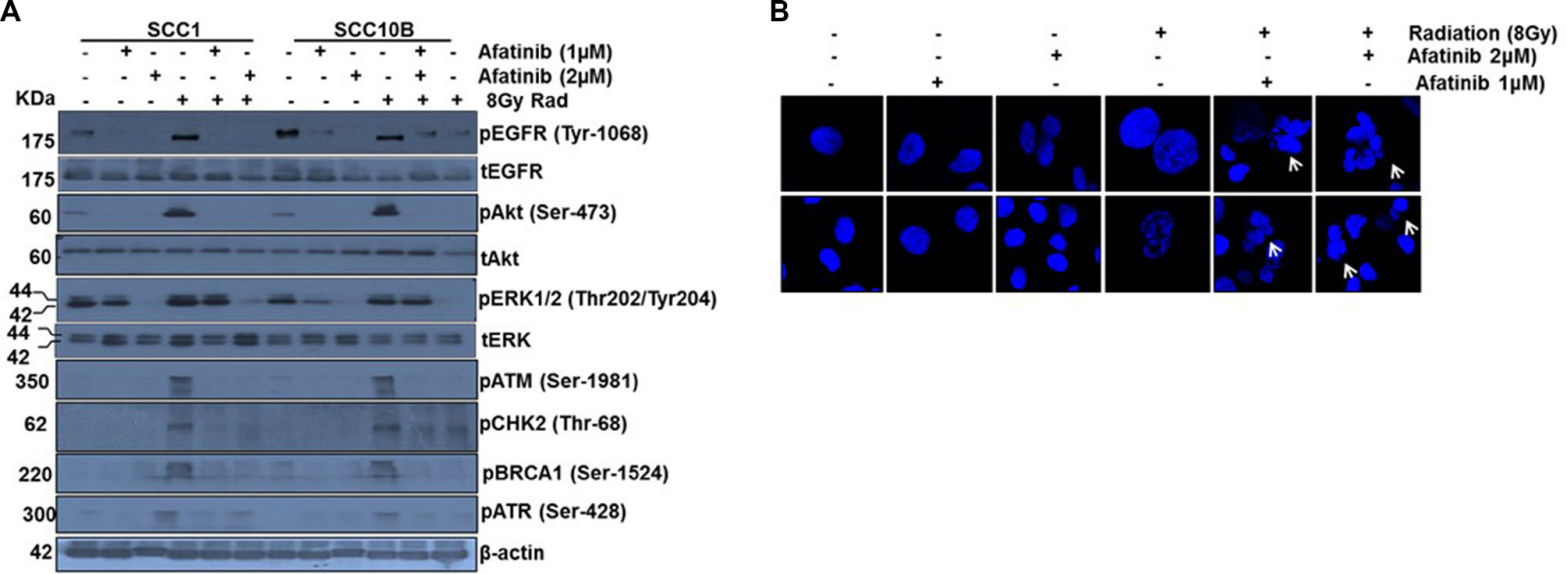
Afatinib attenuates IR-induced DNA repair machinery and induced mitotic catastrophe in HNSCC cells (**A**) HNSCC cells SCC1 and SCC10B were treated with afatinib alone for 48 h, or combined with 8 Gy IR. Cell lysates were analyzed by Western blot analysis for DNA repair pathway proteins including pEGFR (Tyr-1068), pAkt, pERK1/2 (Thr202/Tyr204), pCHK2 (Thr-68), pBRCA1 (Ser-1524), pATM (Ser-1981), and pATR (Ser-428). β-actin was used as an internal loading control. (**B**) SCC1 and SCC10B were treated with afatinib alone for 48 h or combined with 8 Gy IR. Cells were washed and observed under microscope for DAPI staining. The number of cells containing fragmented nuclei (catastrophic nuclei) were photographed.

IR activates EGFR/ATM/ATR/BRCA1/Chk1/Cdc25C-Cdk1/Cyclin B1 signaling cascade (reviewed in [30]) to inhibit G_2_ cell transition and induce cell cycle arrest in S phase for effective DSBs (DNA double strand break) repair which contributes to radio resistance [31]. However, failure of the DNA damaged cells to remain in the S phase due to deficiency of G_2_ checkpoints subjects them to undergo a form of mitotic cell death called mitotic catastrophe (MC), which is characterized by the formation of micronuclei and/or multiple nuclei [27]. We observed a significant upregulation of pEGFR1 upon treatment of SCC1 and SCC10B cells with 8Gy radiation (Figure 4A), and this IR-induced pEGFR1 expression was significantly downregulated when cells were pretreated with afatinib (1–2 μM) for 24 h (Figure 4A), suggesting that afatinib can attenuate IR induced EGFR-mediated DNA repair. Of importance, coupled to the effects on EGFR signaling, we observed increased expression of phosphorylated (activated) Chk2, ATM, ATR, and BRCA1 in 8 Gy-radiated SCC1 and SCC10B cells. Pretreatment with afatinib (1–2 μM) for 24 h significantly inhibited IR induced activation of all these molecules (Figure 4A). No detectable baseline expression of pChk2, pATM, pATR and pBRCA1 was observed in either cell lines (Figure 4A), and these results corroborated the observed inhibition of clonogenic survival in afatinib- and IR-treated SCC1 and SCC10B cells, shown in Figure 2A. We also observed that afatinib pretreatment increased the number of multinucleated cells in irradiated SCC1 and SCC10B cells, compared to only IR-treated cells (Figure 4B). Taken together these results taken together suggest that abrogation of IR-induced DNA DSB repair that leads to MC may in part be one of the mechanisms of afatinib-mediated radio-sensitization of HNSCC.

### Afatinib inhibits radiation-induced side population

**Figure 5:**
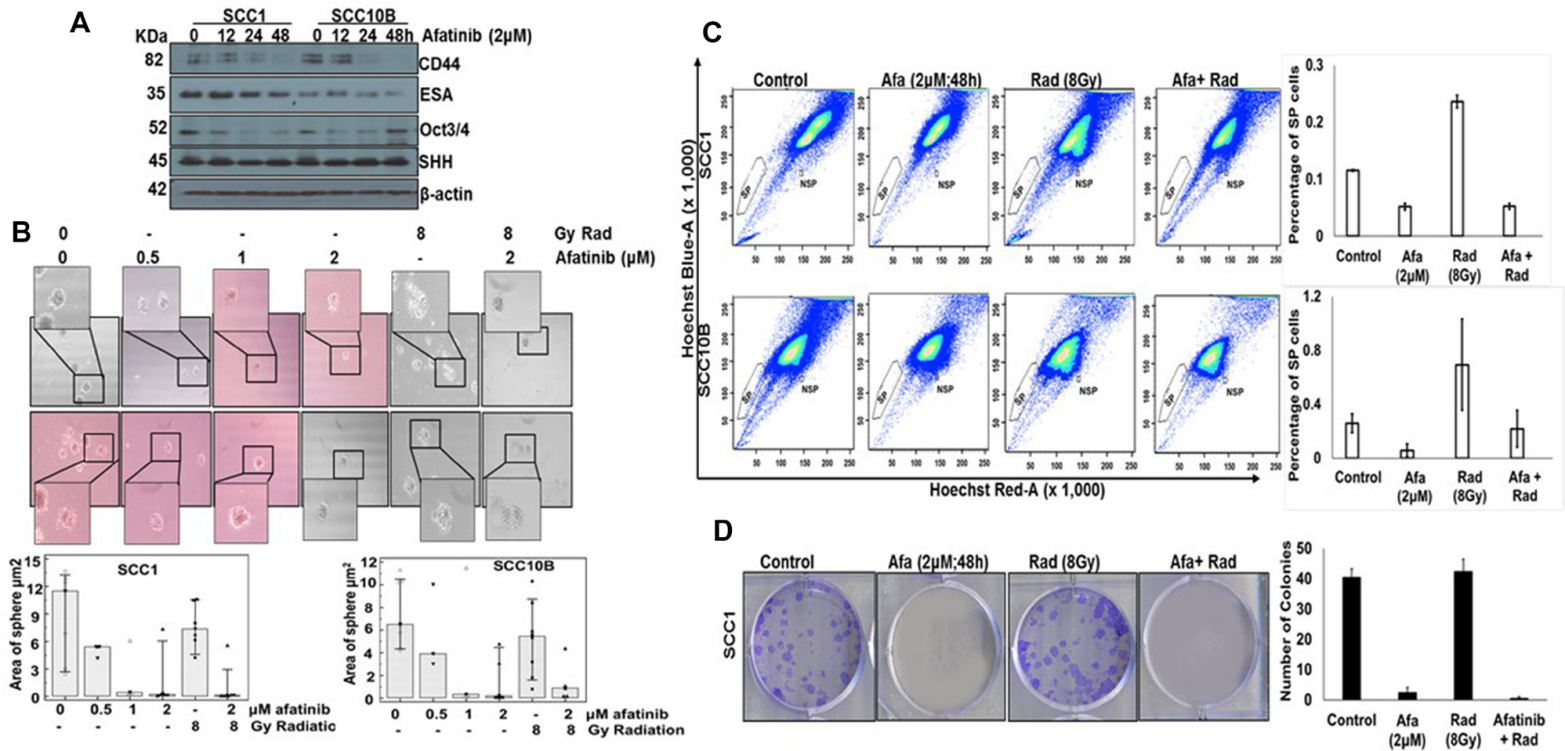
Afatinib affects cancer stem cells in HNSCC cells (**A**) SCC1 and SCC10B cells were treated with 2 μM of afatinib for 12–48 h and analyzed for expression of cancer stem cell markers including CD44, ESA, SHH and Oct3/4 by Western blot analysis. (**B**) SCC1 and SCC10B cells were treated with 0.5–2μM of afatinib alone for 48 h and SP and NSP cells were isolated by FACS analysis using Hoechst 33342 (5 mg/ml) staining. 1 × 10^3^ SP and NSP cells were plated in 24-well low attachment plates and analyzed for sphere formation on 14th day and photographed using light microscope. (**C**) SCC1 and SCC10B cells were treated with either afatinib (48 h) alone or combined with IR. After 48 h, cells were trypsinized and analyzed for SP and NSP cells by FACS analysis using Hoechst 33342 (5 mg/ml) staining. (**D**) Isolated SP cells (250) were plated on 6 well plate and analyzed for colony formation assay after 2 weeks. The graphs represent the mean (± SE) number of colonies. The experiment was repeated twice (*p < 0.05).

SP (or cancer stem cells [CSCs]) is a small population among the majority of tumor cells that displays high tumorigenicity and serves as a reservoir for CRT resistant refractory tumors [32, 33]. EGFR signaling plays an important role in regulating and maintaining cancer stem cells (CSCs) in nasopharyngeal carcinoma [14]. To determine if afatinib interferes with the pathways involved in migration, and self-renewal of CSC cells, we evaluated the effects of afatinib on CSCs by analyzing the expression of CSC markers CD44, Oct4, ESA and SHH. Western blot analysis of afatinib (2 μM)-treated SCC1 and SCC10B cells at various time points showed significant down-regulation of CD44 and Oct3/4 (Figure 5A), suggesting that afatinib effects the self-renewal and invasive properties of CSCs. No change in the expression of SHH and ESA was observed (Figure 5A). To further analyze the effects of afatinib on self-renewal of SP, cells were isolated from afatinib (0.5–2 μM; 48 h)-treated SCC1 and SCC10B cells and analyzed for tumor sphere-forming capability. We observed that afatinib treatment significantly inhibited the formation and growth of tumor spheres formed by SP cells, compared to untreated cells in a dose-dependent manner (Figure 5B).

Recently, IR treatment was shown to enhance the CSC population [15], suggesting that CSCs represent a potential target for improving radiation response in HNSCCs. To investigate the effect of afatinib on radiation-induced CSCs, we treated SCC1 and SCC10B cells with afatinib alone, or in combination with IR, and analyzed for SP and NSP populations. We observed that IR treatment resulted in 2-fold and 2.66-fold enrichment in the SP population in SCC1 and SCC10B cells, respectively, compared to untreated cells (from 0.116% to 0.2365% in SCC1 and from 0.26% to 0.694% in SCC10B cells). However, pre-treatment with afatinib abrogated IR-induced enrichment of SP cells, with only 0.0525% and 0.219% SP cells in SCC1 and SCC10B cells, respectively (Figure 5C). To determine the long-term impact of afatinib on IR enriched SP cells, we analyzed the isolated SP cells for their ability to form colonies and tumor spheres. Afatinib pre-treatment significantly reduced the colony- and tumor sphere-forming ability of IR-enriched SP cells, compared to controls (Figure 5B). Similar results were observed in the colony formation assay (Figure 5D).

### Afatinib radio-sensitizes HNSCC xenografts *in vivo*

**Figure 6:**
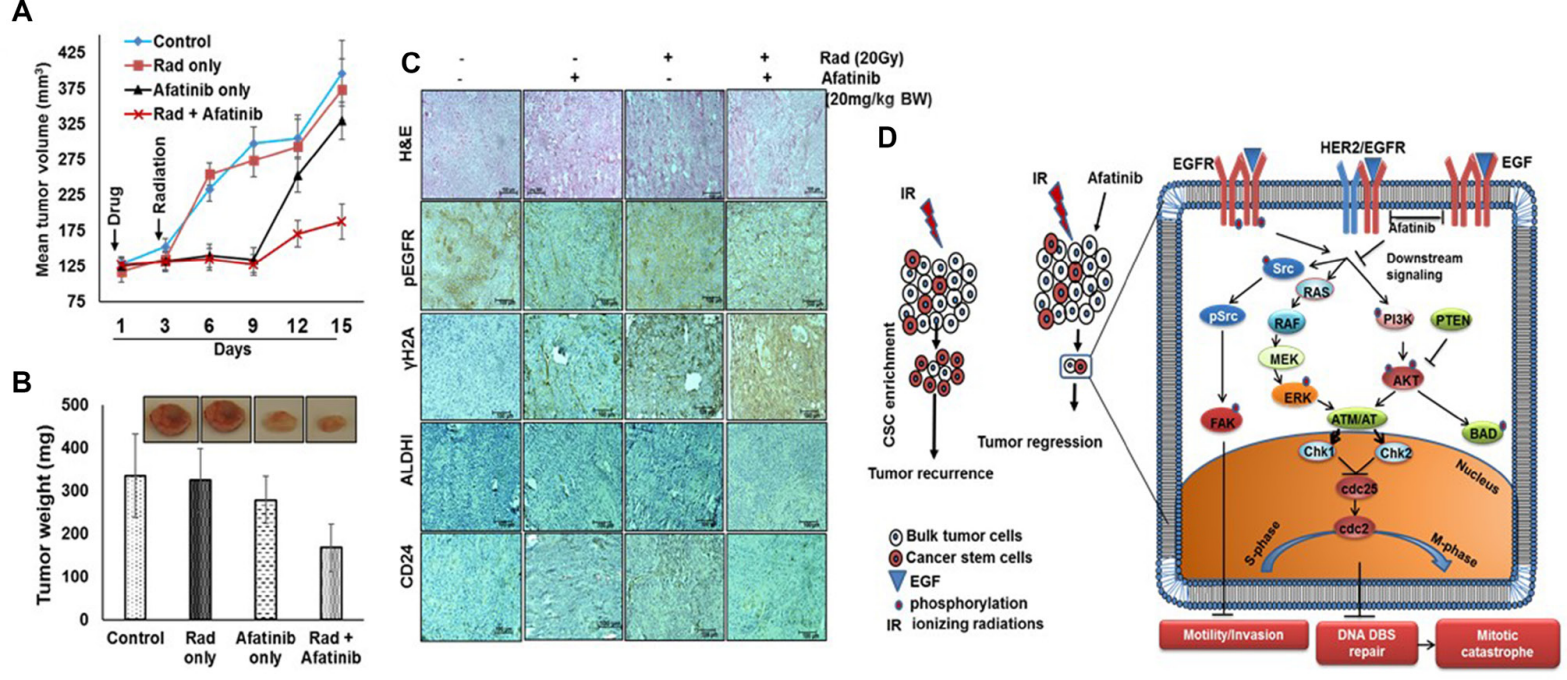
Afatinib radiosensitizes HNSCC tumors *in vivo* (**A**) SCC1 cells were subcutaneously implanted on contralateral flanks in athymic nude mice and randomized into group 1 (8 animals each) with afatinib treatment and radiation on the right tumors, and afatinib only on the left side tumors; group 2 (8 animals each) with vehicle gavages and radiation on the right tumors. Tumor volume and animal weights were measured every 3 days starting from the day of drug administration. All the mice were sacrificed on the 15th day after afatinib treatment and body weight and tumor weight measured. The graphs show a significant decrease in tumor volume (**A**) and tumor weights (**B**) in afatinib + IR treated animals compared to control, afatinib only, or IR-treated mice. (**C**) Excised tumors were analyzed for pEGFR (Tyr-1068), pγH2AX (Ser-139), CD24, and ALDH1 expression using immunohistochemical analysis (×20 magnification). (**D**) Schematic diagram illustrating the potential molecular mechanism of afatinib mediated radio-sensitization of HNSCC. Treatment of ionizing radiation (IR) kills the bulk of tumor cells but enriches cancer stem cells shown as red (left side) that leads to tumor recurrence. However, pre-treatment of afatinib radio-sensitizes tumors and inhibits both CSCs and the bulk of tumor cells, and results in significant tumor shrinkage. The molecular mechanism revealed that afatinib significantly inhibited the phosphorylation of EGFR, HER2, and HER3 coupled with inhibition of downstream signaling molecules, including pAkt (Ser-473) and pERK1/2 (Thr202/Tyr204). Afatinib pre-treatment abrogated IR-induced activation of DNA DSB repair by inhibiting pAkt (Ser-473) and pERK1/2 (Thr202/Tyr204), pATM (Ser-1981), pChk2 (Thr-68), pATR (Ser-428) and pBRCA1 (Ser-1524). In addition, afatinib inhibited IR-induced SP and NSP population and downregulated expression of cancer stem cell markers CD44 and Oct3/4.

To investigate the radio-sentizing effects of afatinib *in vivo*, SCC1 cells were subcutaneously implanted on the contralateral flanks of athymic mice. Three days prior to radiation, mice bearing at least 100 mm^3^ of tumors were randomized to receive either afatinib or vehicle by oral gavages. Tumors on the right flank received a single fraction of 20 Gy IR. Tumor volume and animal weights were measured twice a week for 2 weeks. Afatinib treated or IR treatment alone, resulted in modest inhibition of tumor growth [median weight of 324.914 ± 28.08 mg (IR) and 280.07 ± 20.54 mg (afatinib treated) compared to 335.143 ± 36.67 mg in control tumors. However, tumors that received a combination of afatinib and IR exhibited a significant reduction in tumor volume (168.25 ± 20.85 mg; *p* = 0.05) compared with tumors receiving IR alone (324.91 ± 28.08 mg) (Figure 6A and 6B).

We also evaluated the expression of pEGFR1, pγH2AX, CD24 and ALDH1 in SCC1 tumor xenografts. Higher expression of pEGFR1, CD24, and ALDH1 was observed in radiation-treated tumors, compared to modest staining in untreated tumors and very weak staining in tumors that received either afatinib or combination of afatinib and IR (Figure 6C). While no expression of pγH2AX was detectable in vehicle or afatinib-treated tumors, intense immunostaining was observed in IR-treated tumors. Immunostaining was increased in the tumors treated with afatinib and IR (Figure 6C). These *in vivo* studies corroborated our *in vitro* observations and supported the hypothesis that afatinib inhibits cell proliferation and radio-sensitizes HNSCC tumors.

## DISCUSSION

Radiation treatment, either alone or in combination with chemotherapy, remains the mainstay of treatment for locally advanced (LA) and metastatic HNSCC. However, marginal improvement in the prognosis of LA and metastatic HNSCC patients has been achieved due to intrinsic tumor radio resistance [5–7]. Here we determined the radio-sensitizing potential of a pan-EGFR inhibitor afatinib [20, 21] and explored the mechanism by which afatinib augmented radio-sensitivity in HNSCC cancer models *in vitro* and *in vivo*. Our study showed significant inhibition of EGFR signaling, and this was associated with decreased cell proliferation and significant radio-sensitization of HNSCC cells and tumors caused by afatinib. Our comparative analysis also showed that afatinib was more potent in inhibiting HNSCC cell proliferation and caused less toxicity to normal cells compared to erlotinib (Figure 1A). Overall, our current study suggested a narrow therapeutic window for the efficacy of afatinib as a radio-sensitizer for a subset of HNSCC patients that overexpressed EGFR. Earlier *in vitro* studies demonstrated antiproliferative and radio-sensitizing activity of afatinib [21, 26]. However, these studies were limited due to: (a) use of a single cell line; and (b) no evaluation of toxicity on normal cells. These limitations prompted us to test afatinib in a panel of HNSCC cell lines representing different anatomical subsites and aggressiveness [34], and on normal epithelial cells [35]. We observed that across all tumor cell lines, afatinib more effectively inhibited EGFR activation compared to erlotinib (Figure 1C). This finding supports the utility of afatinib for heterogeneous HNSCC, but furthermore, we made the important observation that afatinib radio-sensitizes HNSCC cells *in vitro* and *in vivo* (Figures 2A and 6A). Of even greater interest, we showed that afatinib inhibited the expression of CSC markers, including CD44 and Oct3/4, and decreased the growth of CSCs. Together, these results demonstrate significant radio-sensitization caused by afatinib is associated with its ability to eradicate CSCs. These studies, therefore, support the utility of afatinib as a radio-sensitizer in the multimodal treatment of HNSCC.

Different phases of the cell cycle respond differently to the cytotoxic effects of radiation, with the S phase being more resistant but G_1_/G_2_-M being most sensitive to IR [36]. Huang et al. have shown that erlotinib radio-sensitizes tumors by decreasing S-phase fraction cells and promoting accumulation of cells in the G_2_-M and G_1_ phases [36]. Our flow cytometry analysis also revealed that treating HNSCC cells with afatinib and IR resulted in G_1_ and G_2_/M phase arrest, respectively, and that treatment reduced the proportion of S-phase cells. Pre-treatment with afatinib followed by IR also further reduced the fraction of S-phase cells. Since IR-treated cells can repair DNA and proliferate normally, reduction of S-phase cells by pre-treatment with afatinib may inhibit the repair of damaged DNA and promote death by apoptosis, cellular senescence, or mitotic catastrophe (MC) [27]. Although we did not observe apoptosis (Figure 2B), our results revealed multinucleated cells in afatinib- and IR-treated cells compared to cells irradiated with IR alone (Figure 4B), and this suggests that induction of MC is one of the mechanisms of radio-sensitization by afatinib.

Epithelial-mesenchymal transition (EMT) is regulated by various transcription factors, including Twist, Snail, Slug, ZEB1, and ZEB2, and are deregulated in many cancers [37]. During EMT, loss of cell adhesion molecules such as E-cadherin results in aggressive tumor behavior [28], CRT resistance [28], and increases CSCs [38]. EGFR activation induces EMT, resulting in resistance to cetuximab or IR in HNSCCs [29] and erlotinib resistance in lung cancers [39]. In addition, EGFR1/Src signaling modulates the FAK/integrin signaling pathway to enhance proliferation, migration, and invasion both *in vitro* and *in vivo* [40] by inducing E-Cadherin internalization [41]. We thus speculated that EGFR mediates EMT by E-cadherin down-regulation and can induce radio-resistance in HNSCC cells. While analyzing the effects of afatinib on EMT, we observed up-regulation of E-cadherin and down-regulation of Snail, Slug, and pFAK, that are associated with decreased motility and invasion (Figure 3A–3C). This observation suggested that induction of mesenchymal to epithelial transition (MET) by upregulating E-cadherin expression may be one of the mechanisms of radio-sensitization of HNSCC cells by afatinib.

Radiation exposure induces DNA double-strand breaks (DSBs) (characterized by the presence of γH2AX foci) that result in apoptosis, cell cycle arrest, and tumor regression [27]. However, in response to IR, some tumor cells activate DSBs repair and pro-survival pathways such as EGFR/ATM/ATR/BRCA1/Chk1 and PI3K/Akt or RAS/Raf/ERK1/2, rendering them radio-resistant (reviewed in [30]). Several studies have shown that afatinib increases IR-induced γH2AX foci in bladder cancer, NSCLC, and pancreatic cancer (PC) cells (reviewed in [42]). These studies suggest that EGFR inactivation by afatinib leads to the inhibition of DSB repair, and to subsequent cell cycle arrest and/or apoptosis. In line with these observations, we noticed increased γH2AX foci in IR-treated cells compared to control and afatinib-treated cells, and afatinib pre-treatment increased IR-induced pγH2AX foci (Figure 2B), which suggests that afatinib inhibits DSB repair. Radio-sensitization induced by afatinib was also associated with a marked decrease in the IR-induced phosphorylation of Akt and ERK1/2, pATM, pATR, pChk1, pBRCA1 and pBRCA2 in SCC1 and SCC10 cells, compared to cells treated with IR (Figure 4A). These results were consistent with our cell cycle analysis data showing a decrease in S-phase cells and an arrest during the G_1_ phase that ultimately contributes to radio-sensitization of cells (Figure 2B). In addition to our *in vitro* findings, we observed significant radio-sensitization of SCC1 xenografts *in vivo* (Figure 6A–6B). There was a significant decrease in tumor growth with a combination afatinib and IR treatment compared to untreated and irradiated SCC1 tumors (Figure 6A–6B). However, in contrast to our *in vivo* data, previous studies reported only marginal radio-sensitization *in vivo* [20, 21]. These apparent differences may attributed to differences in the genetics of the cell lines (FaDu vs. SCC1), or to the mouse backgrounds (SCID vs athymic) used in the two studies.

Cancer stem cells (CSCs) result in tumor recurrence and metastasis and are resistant to chemoradiation therapy [15]. The EGFR family is involved in regulating and maintaining CSCs [14], and recently, Wang et al. have shown that afatinib eliminates CSC populations and inhibits their self-renewal [19]. Our studies also demonstrate that afatinib decreases the expression of CSC markers, including CD44 and Oct3/4 in SCC1 and SCC10B cells, and that it reduced the colony- and tumor sphere-forming capability of CSCs. IR has been shown to enrich CSCs [15]. Irradiation of FaDu cell xenografts showed enhancement of tumor cell population with upregulated EGFR [16], while the combination of cetuximab and IR reduced tumor cells and improved local control [43, 44]. Although these studies did not analyze effects on CSCs, it nevertheless seems logical to speculate that control of local recurrence in cetuximab- and IR-treated tumors may be due to a decrease in CSCs. We analyzed the effect of IR on CSCs and also found a significant increase in the number of CSCs in both SCC1 and SCC10B cells (Figure 5C). Of interest, afatinib compared pre-treatment significantly reduced the proportion of CSCs to untreated cells (Figure 5C). IHC analysis in tumor xenografts also showed decreased expression of the CSC markers CD24 and ALDH1 in afatinib alone and in pre-treated tumors (Figure 6C). Further, afatinib alone and in combination with IR significantly reduced the size of the tumor spheres and decreased the number of colonies obtained from isolated CSCs. These results suggest that combination of afatinib and IR can eradicate CSCs and bulk tumor cells, and may prevent tumor recurrence.

Cisplatin-based CRT is the standard treatment for LA and recurrent HNSCC tumors [45]. Although cisplatin significantly decreases growth and radio-sensitizes HNSCC tumors, both cisplatin and radiation have been shown to enrich the CSC population [15, 46]. While our results show dramatic radio-sensitization of HNSCC *in vitro* and *in vivo* by afatinib and abrogated IR-induced CSC enrichment, our current investigations are focused on the use of afatinib as an adjuvant with CRT to establish a novel treatment regimen with less toxicity and enhanced therapeutic efficacy. We have demonstrated the radio-sensitizing effects of afatinib on HNSCC both *in vitro* and *in vivo*. The possible mechanism of radio-sensitization is schematically illustrated in Figure 6D. However, determining the radio-sensitizing effects of afatinib using athymic mice would not be appropriate, considering the involvement of the tumor microenvironment (TME) in EGFR signaling [47] and that recent observations suggest that irradiation-induced stromal and immunological changes in the TME determine treatment outcomes (reviewed in [48]). Therefore, further studies using spontaneous animal models of HNSCC progression are necessary to analyze the radio-sensitizing effects of afatinib and to understand its mechanism.

## MATERIALS AND METHODS

### Chemicals and antibodies

Afatinib (BIBW2992) and erlotinib (OSI-744) were purchased from Selleck Chemicals (TX, USA). The protein assay kit was from Bio-Rad (Hercules, CA, USA). The E-cadherin antibody was a gift from Dr. Keith R. Johnson (University of Nebraska Medical Center). All other antibodies used are listed in Supplementary Table 1.

### Cell culture

HNSCC cells SCC1, SCC10B (from Dr. Thomas Carey, University of Michigan, Ann Arbor, MI, USA) [34] and immortalized normal oral epithelial cells MOE1a and MOE1b were cultured as previously described [35], and were authenticated by short tandem repeat DNA profiling at the Munroe-Meyer Institute, University of Nebraska Medical Center. MTT assay, Western blot, cell cycle analysis, and confocal microscopy was performed as described previously [34].

### Clonogenic survival assays

Clonogenic survival was performed as described previously [49]. In the radiation survival experiment, cells were treated for 24 h with 0.5 μM/L of afatinib or vehicle control and irradiated with γ-rays (RS 2000 X-RAY irradiator, Rad Source Technologies, Inc., Suwanee GA) with 2–8 Gy/min [34, 49]. After an additional 24 h, cells were washed and grown in drug-free medium for 14 days. Cells were stained with 0.5% crystal violet; colonies with ≥ 50 cells were counted. Plating efficacy and survival fractions were calculated and cell survival curves were fitted using the least squared regression by the linear quadratic model, using Sigmaplot^™^ 10.0 software (Systat Software, Inc., Chicago, IL, USA) [49]. The sensitization enhancement ratio (SER) was calculated as the dose needed to produce 10% cell survival for IR alone divided by the dose to achieve such using IR plus afatinib [34]. SER values >1.0 indicates enhancement of radio-sensitivity.

### Isolation of SP cells and spheroid formation assay

SP and NSP cells were isolated as previously described [19]. For spheroid formation assay, both SP and NSP cells (1 × 10^3^ cells/ml) were cultured on ultra-low attachment plates with serum-free DMEM/F12 medium supplemented with 10 ng/ml fibroblast growth factor, 20 ng/ml epidermal growth factor, 10 ng/ml basic fibroblast growth factor (bFGF), and 5 mg/ml insulin. The spheroid formed were evaluated after 7–14 days of culture and spheroid size were measured using Motic Image Plus 2.0 software (Motic Asia, Hong Kong) and represented as μm^2^. For colony formation assay, 150 SP cells were seeded on 6 well plates and analyzed as described previously [49].

### Subcutaneous xenografts and immunohistochemistry

*In vivo* studies were performed according to the UNMC Institutional Animal Care and Use Committee (IACUC). Exponentially growing SCC1 (0.5 × 10^6^) cells in 50 μl PBS were implanted subcutaneously into the right and left hind flank of athymic mice [49]. Ten days after implantation, tumors were measured using Vernier calipers and animals were randomized to receive vehicle, afatinib (20 mg/kg/day), radiation (20 Gy) and afatinib plus radiation. Tumors were CT scanned and one fraction of 20 Gy radiation was delivered stereotactically as described by us [49]. Three days before irradiation, mice received afatinib by oral gavages in 100 μl of H_2_O [19] and continued for 1 week. Immunohistochemical analysis of pEGFR1, CD24, ALDH1, and pγH2AX in histologic sections were determined [34, 50].

### Statistical analysis

The data was analyzed using the Medcalc software (version 9.6.4.0) for windows. The independent-sample *t-test* was used to analyze the treatment response of tumor allografts. The criterion for statistical significance was *P < 0.05, **P < 0.01 and ***P < 0.005.

## SUPPLEMENTARY MATERIALS TABLES



## Abbreviations

HNSCC: Head and neck squamous cell carcinoma
OSCC: Oral squamous cell carcinoma
EGFR: Epidermal growth factor receptor
IR: Ionizing radiation
LA: Locally advanced
CRT: Chemoradiation Therapy
TKIs: Tyrosine Kinase Inhibitors.
