# Supplementary material for: Afatinib radiosensitizes head and neck squamous cell carcinoma cells by targeting cancer stem cells
Source: Oncotarget. 2017 Feb 18;8(13):20961–73. doi: 10.18632/oncotarget.15468 (PMC5400558; doi:10.18632/oncotarget.15468)
Supplement: Supplementary file 1 [file oncotarget-08-20961-s001.pdf]

# Afatinib radiosensitizes head and neck squamous cellcarcinoma cells by targeting cancer stem cells

## Supplementary Materials

**Supplementary Table 1: List of antibodies used in the study**

| S.No | Antibody Catalog | Catalog | Vendor | Dilution |
|---|---|---|---|---|
| 1 | EGFR (D38B1) | MA#4267 | Cell Signaling Technology | 1:1000 |
| 2 | pEGFR (Y1068) | #3777 | Cell Signaling Technology | 1:1000 |
| 3 | tHER2 | SC-134481 | Santa Cruz Biotechnology | 1:1000 |
| 4 | pHER2(Y1248) | #2247S | Cell Signaling Technology | 1:1000 |
| 5 | tHER3 | SC-8050 | Santa Cruz Biotechnology | 1:1000 |
| 6 | tHER4 | SC-285 | Santa Cruz Biotechnology | 1:500 |
| 7 | pAkt (S473) | #4060X | Cell Signaling Technology | 1:1000 |
| 8 | AKT | #4691L | Cell Signaling Technology | 1:1000 |
| 9 | ERK1/2 (MK1) | SC-135900 | Santa Cruz Biotechnology | 1:500 |
| 10 | p-ERK1/2 (T202/Y204) | #9101L | Cell Signaling Technology | 1:1000 |
| 11 | pFAK (Y397) | SC11765-R | Santa Cruz Biotechnology | 1:1000 |
| 12 | pP38 MAPK (Thr-108/Tyr-182) | #9211 | Cell Signaling Technology | 1:1000 |
| 13 | pP38 MAPK | #9212 | Cell Signaling Technology | 1:1000 |
| 14 | pATM (Ser-1981) | #9947S | Cell Signaling Technology | 1:1000 |
| 15 | pCHK2 (Thr-68) | #9947S | Cell Signaling Technology | 1:1000 |
| 16 | pBRCA1 (Ser-1524) | #9947S | Cell Signaling Technology | 1:1000 |
| 17 | pATR (Ser-428) | #9947S | Cell Signaling Technology | 1:1000 |
| 18 | PγH2A (Ser-139) | #9947S | Cell Signaling Technology | 1:1000 |
| 19 | ZO-1 | #9782S | Cell Signaling Technology | 1:1000 |
| 20 | Snail | #9782S | Cell Signaling Technology | 1:1000 |
| 21 | Slug | #9782S | Cell Signaling Technology | 1:1000 |
| 22 | pFAK (Tyr-925) | #3284 | Cell Signaling Technology | 1:1000 |
| 23 | tFAK | SC-271195 | Santa Cruz Biotechnology | 1:1000 |
| 24 | CyclinD1 | SC-753 | Santa Cruz Biotechnology | 1:1000 |
| 25 | Cyclin A | SC-751 | Santa Cruz Biotechnology | 1:1000 |
| 26 | Cyclin B1 | SC-594 | Santa Cruz Biotechnology | 1:1000 |
| 27 | ALDHI/2 | SC-50385 | Santa Cruz Biotechnology | 1:1000 |
| 28 | CD24 | SC-11406 | Santa Cruz Biotechnology | 1:1000 |
| 29 | β-actin | A2228 | Sigma-Aldrich | 1:5000 |
